# Transarterial Chemoembolization Modulates the Exosomal miR-32-5p/cGAS-STING Axis Mediated Macrophage Ferroptosis, Triggers Immune Remodeling, and Enhances Anti-PD-1/L1 Efficacy in HCC

**DOI:** 10.34133/research.1096

**Published:** 2026-01-27

**Authors:** Bufu Tang, Dandan Guo, Weiliang Hou, Wen Zhang, Wei Zhang, Jingqin Ma, Changyu Li, Guowei Yang, Lin Zhu, Min Li, Xuran Jin, Zhiping Yan, Qianqian Zhao, Yongjie Zhou, Xudong Qu

**Affiliations:** ^1^ Department of Interventional Radiology, Zhongshan Hospital, Shanghai Institute of Medical Imaging, National Clinical Research Center of Interventional Medicine, Fudan University, Shanghai, China.; ^2^ Department of Oncology, The First Affiliated Hospital of Dalian Medical University, Dalian 116011, China.; ^3^ Department of Gastroenterology, Shanghai Institute of Pancreatic Diseases, National Key Laboratory of Immunity and Inflammation, Changhai Clinical Research Unit, Changhai Hospital, Naval Medical University, Shanghai, China.; ^4^ Department of Radiation Oncology, Zhongshan Hospital, Fudan University, Shanghai, 200032, China.

## Abstract

Hepatocellular carcinoma (HCC) is a cancer type that causes a high rate of cancer death in the world. The standard therapy plan of intermediate and advanced stages of the HCC is transarterial chemoembolization (TACE). The treatment effectiveness is, however, limited because of the heterogeneity of tumors and the resistance to drugs. This paper shows that the HCC patients with TACE resistance alter their tumor immune homeostasis by reducing the secretion of exosomal miR-32-5p, which has a negative relationship with the population of CD68^+^ macrophages. Both in-cellular and animal studies show that exosomal miR-32-5p leads to ferroptotic cell death in tumor-associated macrophages (TAMs) characterized by augmented lipid oxidation, iron buildup, depletion of glutathione, and mitochondrial malfunction. At the same time, miR-32-5p increases production of M1-type proinflammatory factors such as CD86, CCL2, tumor necrosis factor-α (TNF-α), and interleukin-6 (IL-6), thereby enabling macrophage polarization toward tumor-suppressive phenotype. Mechanistically, miR-32-5p activates the cyclic GMP-AMP synthase (cGAS)–stimulator of interferon genes (STING) signaling pathway through ARID1B down-regulation, ultimately remodeling the tumor immune microenvironment. Experimental murine models indicated that the delivery of exosomal miR-32-5p was a strong tumor suppressor and disseminator, increased the recruitments of CD86 + antigen-presenting cells and CD8 + T lymphocytes, and boosted anti-neoplastic immunity. It should be highlighted that exosomal miR-32-5p also increased the levels of PD-L1, which reflected its complementary value to anti-PD-L1 immunotherapy. Such a combined treatment led to excellent tumor control and enhanced survival without loss of acceptable toxicity profiles. The essential role of ferroptosis was confirmed by the use of Fer-1 to inhibit the chemical reactions, which revealed a new approach by which TACE-resistant exosomal miR-32-5p could inhibit the progression of HCC and complement the anti-PD-L1 therapeutic effects through ferroptosis using TAM, providing insights as well as potential therapeutic objectives in the treatment of HCC.

## Introduction

Hepatocellular carcinoma (HCC) represents a substantial cause of cancer-associated morbidity and mortality on a global scale. Some of the known treatment regimens used in the treatment of HCC include liver transplant, excision of tumors, and systemic interventions. However, because of high heterogeneity of tumors, which are inherent to HCC, less than optimal clinical outcomes are often observed, particularly in the middle and end stages of cancers, where tumor recurrence and metastases have substantial effect on long-term survival rates [1,2]. Although transarterial chemoembolization (TACE) is standard therapeutic regimen of intermediate to advanced HCC, incomplete obstruction of the arteries during TACE could result in angiogenesis caused by hypoxia and thus could decrease the effectiveness of treatment and affect patient outcome adversely [3–5]. Emerging evidence underscores the therapeutic potential of ferroptosis modulation in HCC. Elucidating the molecular crosstalk between ferroptosis and HCC progression may inform the design of novel interventions to enhance treatment outcomes.

Ferroptosis, characterized by iron-catalyzed lipid peroxidation, has garnered increasing attention as a target in cancer therapy [6–10]. TAMs, as key orchestrators of the immunosuppressive tumor microenvironment (TME), are increasingly recognized as critical determinants of ferroptosis sensitivity and therapeutic outcomes [9]. Previous studies have demonstrated that cystine/glutamate antiporter (xCT) deficiency in macrophages inhibits M2 polarization, triggers ferroptosis, suppresses HCC growth, and prevents metastasis [11]. Therefore, exploring the relationship between ferroptosis and the TME holds considerable potential for developing novel anticancer approaches. The treatment of cancer immunotherapy is gaining vast experimental and clinical support on the difficulty of the TME. Ferroptosis and the TME can interdepend and influence each other through complicated feedback loops and regulation pathways as tumor develops [12–14]. TAMs are among the most abundant immune cells in the TME and are well recognized for their role in supporting tumor progression, from immune suppression to angiogenesis and metastatic spread. Given their critical role in establishing an immunosuppressive TME, TAMs represent valuable targets for immunotherapy [15,16]. TAMs are targets of critical importance in tumor development and are therefore valuable subjects to the new therapeutic interventions in the future.

The exosomes play decisive roles in TME regulation, and the vesicles demonstrate specific significance in changing the macrophage functions and phenotypic characteristics. As fundamental mediators of intercellular communication, exosomes carry diverse molecular cargoes, including proteins, lipids, mRNAs, miRNAs, and DNA fragments [17]. The significance of exosomes in HCC is multifaceted, encompassing roles in tumor development, disease progression, therapeutic resistance, and response monitoring. Additionally, exosomes have emerged as promising biomarkers for tumor detection, prognostic evaluation, and therapeutic monitoring [18,19]. Moreover, the development of molecular components that are delivered via exosomes can support cancer advancement, metastasis, and resistance to drugs through the coordination of cellular communication in the TME. Notably, miR-32-5p plays a central role in regulating multiple malignancies. Most importantly [20,21], there is an emerging literature that indicates that miRNA would impact macrophages that exist in the TME. Such effects are possible through exosome-mediated delivery systems. These processes can facilitate malignant properties of HCC. Therefore, exploring such phenomena is the main goal of the current study.

The current research illuminated the action of exosomal miR-32-5p in the regulative mechanisms of the TME, in modifications of immune reactions, and in the inhibition of cancer development. Systematic mechanisms of exosome-mediated tumor growth were studied, the mechanisms of immune evasion were evaluated, and ferroptosis regulatory circuits were studied. Moreover, the interactions of ferrostatin-1 (Fer-1) as an anti-ferroptotic agent, inhibition of PD-L1, and effects of the interrelations on exosome-mediated cancer enhancement in tumors were also evaluated through experiments, which provide theoretical insights into the research. Potential therapies are identified on the basis of the results, including possible clinical indicators that can be used to support cancer immunotherapy.

## Results

### Exosomal miR-32-5p exhibits tight correlation with macrophage dynamics and post-TACE microenvironment in HCC

**Fig. 1.**
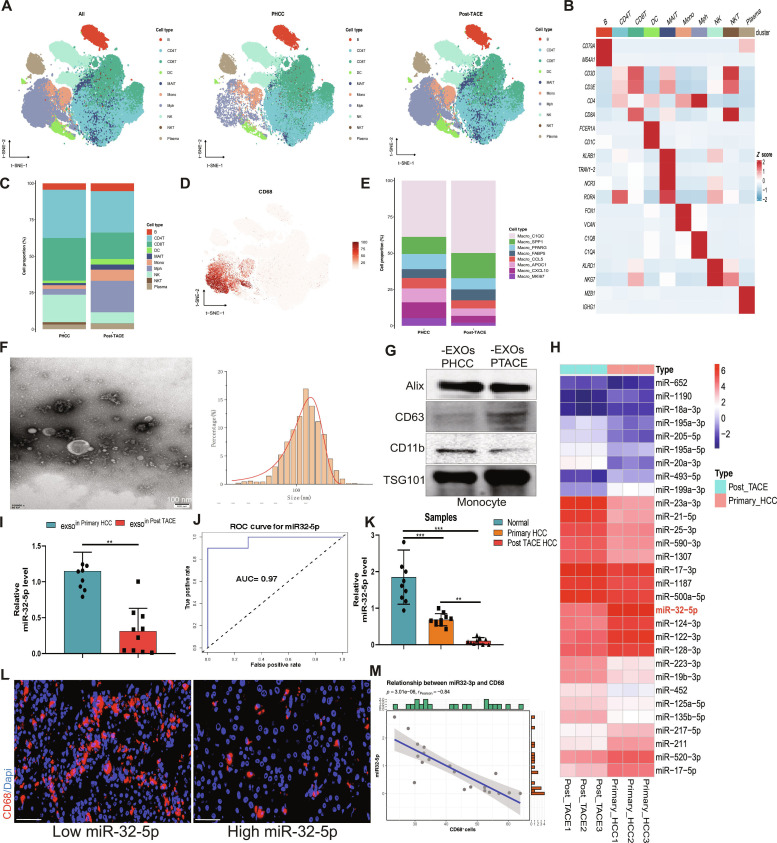
Exosomal miR-32-5p contributes to the remodeling of the TACE immune microenvironment. (A) Uniform Manifold Approximation and Projection (UMAP) visualization depicting the distribution of various cell types in single-cell transcriptomics. (B) Heatmap presenting specific marker genes for individual cell types. (C) Stacked bar chart demonstrating the proportion of cell types among distinct sample groups. (D) UMAP plot revealing the expression distribution of the CD68 gene throughout cell populations. (E) Alterations in the proportion of macrophage subpopulations between PHCC and PTACE samples. (F) TEM image of exosome vesicle structure and nanoparticle tracking analysis (NTA) demonstrating the size distribution of exosomes. Scale bar, 100 nm. (G) Immunoblot detection of exosome marker proteins Alix, CD63, and Tsg101. (H) Expression profiles of miRNAs across different immune cell subpopulations. (I) miR-32-5p expression levels in PHCC and PTACE samples. (J) ROC curve analysis of miR-32-5p as a predictive biomarker for TACE efficacy. (K) Comparison of miR-32-5p expression levels among various clinical sample groups (normal, primary HCC, post-TACE HCC). (L) Immunofluorescence images of CD68 cells in tumor tissues from low-expression and high-expression miR-32-5p groups. (M) Correlation analysis between miR-32-5p expression levels and the proportion of CD68^+^ cells. Scale bar, 20 μm. *P < 0.05; **P < 0.01; ***P < 0.001.

Single-cell transcriptomic study helped to specify the peculiarities of the HCC immune microenvironment, depending on the conditions of treatment [22,23] (Fig. 1A and B). Major changes in the cellular composition were noted between the primary and post-TACE HCC tissues, including a critical difference in the proportion of the different cell populations after treatment with TACE (Fig. 1C to E and Fig. S1A and B). In order to examine the molecular processes, monocyte-derived exosomes were purified and identified. Transmission electron microscopy (TEM) and Western blot (WB) analysis had established the normal exosome morphology and markers (CD63, TSG101) (Fig. 1F and G). miRNA profiling of monocyte-derived exosomes found that there were unique patterns of differentiation between primary hepatocellular carcinoma (PHCC) and post-pransarterial chemoembolization (PTACE) patient samples (Fig. 1H). Interestingly, miR-32-5p expression in monocyte-derived exosomes was significantly decreased in TACE-resistant HCC patients compared to primary HCC patients (Fig. 1I). The receiver operating characteristic (ROC) curve analysis indicated how well exosomal miR-32-5p could effectively diagnose TACE-resistant conditions (Fig. 1J) and confirmed the reduced level of this miRNA in patients with resistance to TACE (Fig. 1K). We next investigated the relationship between miR-32-5p and the TME. Immunofluorescence analysis showed that tissues with low miR-32-5p expression harbored a higher density of CD68^+^ macrophages (Fig. 1L), and correlation analysis confirmed an inverse association between miR-32-5p levels and macrophage infiltration (Fig. 1M). These results suggest that exosomal miR-32-5p may serve as a regulator of macrophage dynamics within the HCC microenvironment following TACE treatment.

### Exosome enhances macrophage ferroptosis and activates proinflammatory response via miR-32-5p

**Fig. 2.**
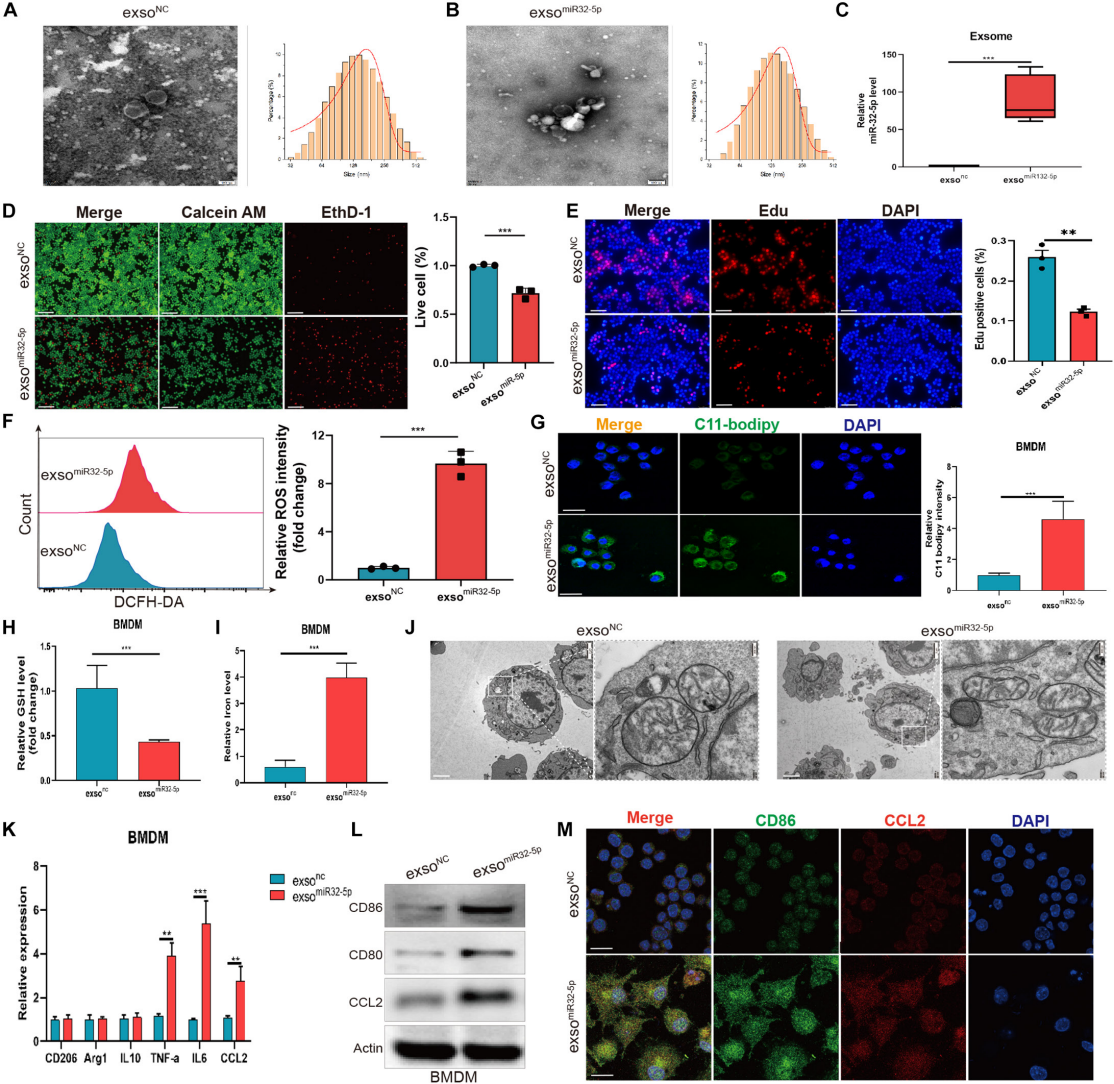
Exosomal miR-32-5p induces ferroptosis and M1 polarization of TAMs. (A and B) TEM images and size distribution analysis of exosomes from exso^NC^ and exso^miR-32-5p^ groups. Scale bar, 100 nm. (C) Measurement of miR-32-5p expression levels in exosomes. (D) Cell viability evaluation of exso^NC^ and exsomiR-32-5p groups using Calcein AM and EthD-1 staining. (E) Representative immunofluorescence micrographs of 5-ethynyl-2′-deoxyuridine (EdU) incorporation in exso^NC^ and exsomiR-32-5p groups. (F) Flow cytometric analysis of lipid peroxide levels in TAMs and their relative fluorescence intensity. (G) Representative C11-BODIPY staining showing lipid peroxidation levels in exso^NC^ and exsomiR-32-5p groups. (H and I) GSH and ferrous ion levels in BMDMs treated with exso^NC^ and exsomiR-32-5p exosomes. (J) TEM analysis of mitochondrial ultrastructure in BMDMs treated with exso^NC^ and exsomiR-32-5p exosomes. (K) Reverse transcription quantitative PCR (RT-qPCR) analysis of M1 and M2 macrophage marker genes CD206, Arg1, IL10, TNF-α, IL6, and CCL2. (L) WB analysis of M1 macrophage-associated proteins CD86, CD80, and CCL2. (M) Immunofluorescence analysis of M1 marker CCL2 and CD86 expression and colocalization. Scale bar, 20 μm. *P < 0.05; **P < 0.01; ***P < 0.001.

To clarify the antitumoral role of miR-32-5p-enriched exosomes, bone marrow-derived macrophages (BMDMs) stably expressing miR-32-5p were generated, from which exso^miR-32-5p^ and exso^NC^ were isolated and validated by standard characterization methods (Fig. 2A to C), confirming substantial miR-32-5p enrichment in these vesicles. When BMDM-derived TAMs were treated with these exosomes, the exso^miR-32-5p^ group exhibited markedly decreased cell viability relative to controls, as evidenced by live/dead cell staining and viability assays (Fig. 2D and E). Moreover, exso^miR-32-5p^ treatment increased cellular lipid peroxidation levels and intracellular ferrous ion concentrations while decreasing glutathione (GSH) levels (Fig. 2F to I). Consistent with these biochemical changes, TEM revealed extensive mitochondrial structural damage in the exso^miR-32-5p^ treatment group (Fig. 2J). Collectively, these ferroptosis-associated changes indicate that miR-32-5p-containing exosomes effectively induce ferroptosis in HCC-associated macrophages. Additionally, exosome treatment markedly elevated the expression of M1 proinflammatory markers (CD86, CCL2, TNF-α, IL-6) in macrophages. Immunofluorescence and confocal microscopy analyses verified the augmented expression and modified colocalization patterns of these proinflammatory molecules (Fig. 2K to M). This study demonstrates that miR-32-5p-containing exosomes regulate HCC-associated macrophage survival through ferroptosis induction while promoting macrophage reprogramming toward a proinflammatory phenotype.

### Exosomal miR-32-5p suppresses tumor growth and metastatic spread and stimulates antitumor immune responses

**Fig. 3.**
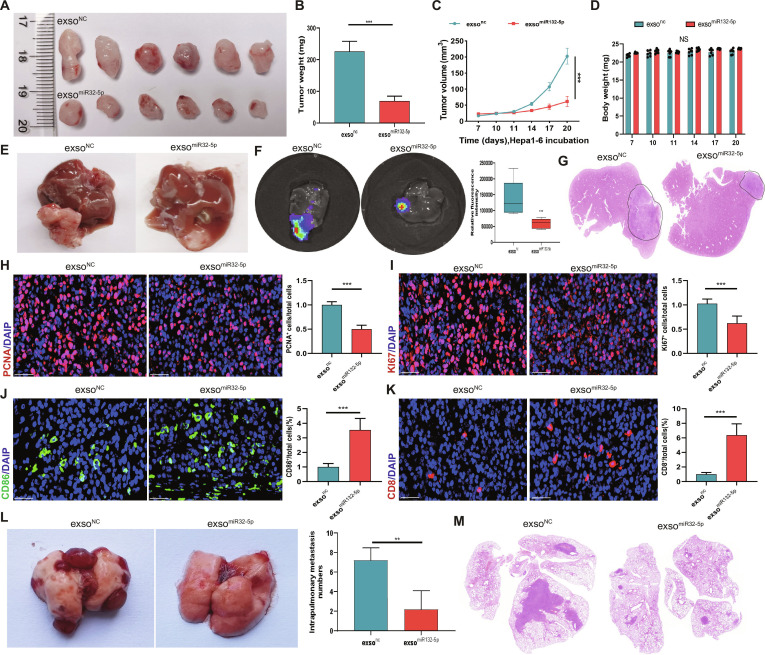
Exosomal miR-32-5p facilitates tumor suppression in HCC through microenvironmental reprogramming. (A) Representative macroscopic images of resected tumors from experimental groups, demonstrating a substantial decrease in tumor size following exso^miR-32-5p^ treatment. (B) Quantitative analysis of tumor weights showing significant reduction in the exso^miR-32-5p^ treatment group compared to exso^NC^ controls. (C) Temporal analysis of tumor growth kinetics revealing significantly reduced tumor progression in the exso^miR-32-5p^ group. (D) Quantitative assessment of body weight changes across treatment groups, indicating minimal systemic toxicity. (E) In the orthotopic HCC model established by intrahepatic implantation, gross pathological examination of tumor specimens showed reduced tumor mass and decreased vascular density in exso^miR-32-5p^-treated samples. (F) In vivo bioluminescence imaging analysis showing reduced tumor burden in exso^miR-32-5p^-treated animals. (G) Histopathological analysis by H&E staining revealing enhanced necrotic areas within tumors from the exso^miR-32-5p^ treatment group. (H and I) Immunofluorescence staining revealing the expression of PCNA- and Ki67-positive cells in the exso^miR-32-5p^ group. (J and K) Immunofluorescence analysis demonstrating the expression of CD86- and CD8-positive cells in the exso^miR-32-5p^ group. (L) In the setting of liver cancer lung metastasis, the exso^miR-32-5p^ group exhibited a significantly decreased number of metastatic lymph nodes compared to the exso^NC^ group. (M) Histological assessment of metastatic lesions by H&E staining showing reduced metastatic spread in exso^miR-32-5p^-treated specimens. Scale bar, 20 μm. *P < 0.05; **P < 0.01; ***P < 0.001.

A xenograft model was developed in vivo to investigate the therapeutic impact of exosomal miR-32-5p in tumor progression and metastasis. Exso^miR-32-5p^ treatment led to strong tumor growth suppression, as evidenced by significant reductions in tumor volume and weight change compared to exso^NC^ controls (Fig. 3A to C). The long-term observation of the body weight did not find any critical differences between the treatment groups, which indicates low systemic toxicity (Fig. 3D). Exso^miR-32-5p^ is also significantly able to reduce the intrahepatic primary tumor burden in the orthotopic HCC model, where hepatic tumor nodules were small in size when assessed grossly (Fig. 3E) and reduced bioluminescence signal (Fig. 3F). Histological examination revealed that there was a smaller tumor area and less lesions in the liver parenchyma, which was further proven by use of quantitative morphometric analysis that indicated that the proportion of intrahepatic tumor area was reduced (Fig. 3G). An immunofluorescence proliferation marker analysis showed that PCNA and Ki67 cells were reduced significantly in exso^miR-32-5p^-treated tumor cells (Fig. 3H and I), which demonstrated inhibited tumor cell proliferation. Moreover, the exso^miR-32-5p^ treatment promoted the infiltration of CD86 + antigen-presenting cells and CD8 + cytotoxic T cell lymphocytes into the TME (Fig. 3J and K), indicating the promotion of antitumor immunity. Interestingly, the exso^miR-32-5p^-treated group showed considerably low rate of pulmonary metastasis, which was confirmed by the histopathological examination (Fig. 3L and M). The above observations reveal that exosomal miR-32-5p suppresses tumor growth and metastasis, and at the same time stimulates antitumor immune responses in the TME.

### miR-32-5p inhibits tumor growth by regulating macrophage ferroptosis and antitumor polarization

**Fig. 4.**
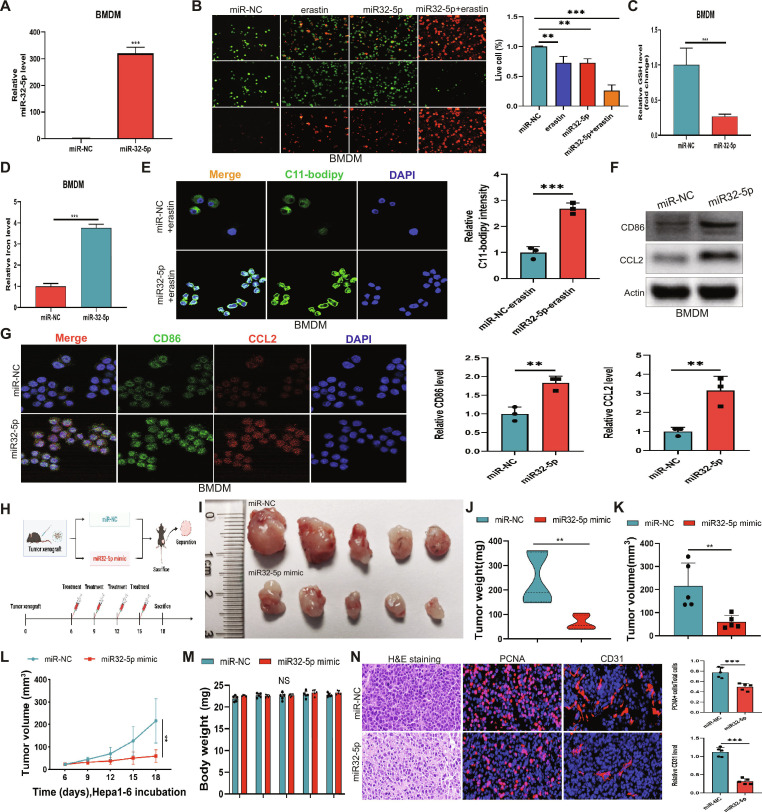
miR-32-5p regulates macrophage polarization and suppresses tumor growth in vivo. (A) Comparative expression profiles of miR-32-5p in BMDMs. (B) Characteristic live/dead staining fluorescence photographs illustrating the impacts of miR-NC, erastin, miR-32-5p, and miR-32-5p + erastin interventions on BMDMs. (C and D) Quantification of GSH and ferrous ion concentrations in BMDMs following miR-NC and miR-32-5p transfection. (E) Immunofluorescence detection of C11-BODIPY labeling in BMDMs following miR-NC or miR-32-5p transfection. (F) WB detection of CD86 and CCL2 protein levels in BMDMs following miR-NC or miR-32-5p transfection, utilizing Actin as internal reference. (G) Immunofluorescence visualization of CD86, CCL2, and nuclear labeling in BMDMs following miR-NC or miR-32-5p transfection. (H) Schematic diagram depicting the experimental animal system. (I) Characteristic gross tumor photographs obtained from miR-NC and miR-32-5p mimic cohorts. (J and K) Statistical analysis of tumor mass (J) and size (K) in miR-NC and miR-32-5p mimic cohorts. (L) Tumor progression kinetics in miR-NC and miR-32-5p mimic cohorts across 18 d following Hepa1-6 implantation. (M) Body mass monitoring demonstrating similar measurements across miR-NC and miR-32-5p cohorts, confirming treatment safety. (N) Characteristic photographs of H&E labeling, PCNA immunolabeling, and CD31 immunofluorescence within tumor specimens obtained from miR-NC and miR-32-5p mimic cohorts. *P < 0.05; **P < 0.01; ***P < 0.001.

To investigate the role of miR-32-5p in regulating tumor growth and macrophage metabolism, both in vitro and in vivo experiments were performed. Transfection of miR-32-5p mimics into BMDMs resulted in significant up-regulation of miR-32-5p expression (Fig. 4A). Fluorescence microscopy showed high level of cell death in miR-32-5p-treated cells compared to miR-NC-treated controls, particularly together with erastin (Fig. 4B). miR-32-5p treatment significantly elevated intracellular iron levels and a significant decrease in GSH (Fig. 4C and D). Further validation of C11-BODIPY staining also supported the occurrence of more lipid peroxidation in miR-32-5p-treated cells (Fig. 4E). WB analysis and immunofluorescence staining indicated that the proteins of CD86 and CCL2 were up-regulated (Fig. 4F and G). In the in vivo tumor model, miR-32-5p mimic treatment significantly reduced tumor weight and volume compared to miR-NC controls (Fig. 4H to K), with consistent growth kinetics observed throughout the experimental period (Fig. 4L). It is important to emphasize that there was no significant systemic toxicity of the treatment, as all the groups had similar body weights (Fig. 4M). Histological analysis revealed that the miR-32-5p-treated tumors had lower cell density and proliferation and lower levels of angiogenesis (Fig. 4N), which means that miR-32-5p inhibits tumor growth via a complex of metabolic reprogramming and inflammatory control.

### Macrophage-specific knockout of miR-32 promotes tumor growth and reshapes the TAM phenotype

**Fig. 5.**
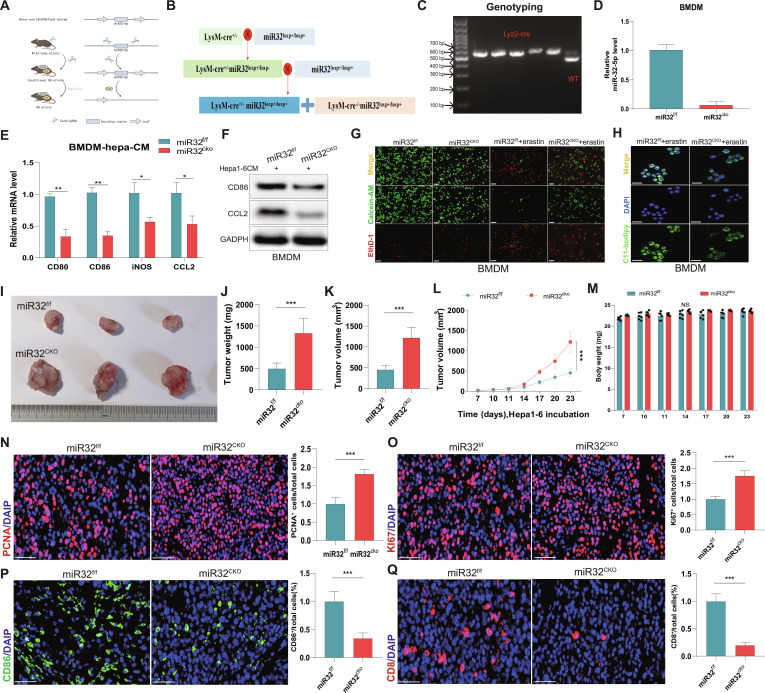
Macrophage-specific deletion of miR-32 accelerates tumor progression and alters TAM phenotype. (A) Schematic representation of the experimental mouse model establishment. (B) Breeding strategy for miR-32-5p transgenic and LysM-cre macrophage-specific deletion mice. (C) Genotype verification of miR-32-5p^CKO^ mice. (D) miR-32-5p expression analysis in BMDMs, showing significant reduction in the miR-32 deletion group. (E) Quantitative analysis of inflammatory gene expression (CD80, CD86, iNOS, CCL2) in BMDMs following HCC cell coculture. (F) WB evaluation of CD86 and CCL2 expression in miR-32^f/f^ and miR-32^CKO^ groups. (G) Assessment of cellular viability following various treatments. (H) C11-BODIPY fluorescence analysis of cellular lipid peroxidation in miR-32^f/f^ and miR-32^CKO^ groups. (I) Representative tumor images from miR-32^f/f^ and miR-32^CKO^ mice. (J and K) Quantitative assessment of tumor weight (J) and volume (K) across experimental groups. (L) Temporal analysis of tumor growth kinetics. (M) Body weight trajectories of tumor-bearing mice. (N) PCNA immunofluorescence analysis with quantitative assessment of tumor cell proliferation. Representative images were obtained from the central tumor region. (O) Ki67 immunofluorescence staining with quantitative analysis. Representative images were obtained from the central tumor region. (P) CD86 immunofluorescence analysis with quantification of positive cells. Representative images were obtained from the central tumor region. (Q) CD8 immunofluorescence assessment with quantification of positive cells. Representative images were obtained from the central tumor region. Scale bar, 20 μm. *P < 0.05; **P < 0.01; ***P < 0.001, NS: not significant.

To further explain the antitumor role of up-regulating miR-32 in macrophage regulation, myeloid cell-specific miR-32^CKO^ mice had been produced by selective breeding of miR-32^f/f^ LysM-Cre mice with miR-32^f/f^ mice (Fig. 5A and B). Genotyping confirmed successful knockout in miR-32^CKO^ mice (Fig. 5C). The expression level of miR-32-5p in BMDM was significantly reduced in miR-32^CKO^ mice compared to miR-32^f/f^ mice (Fig. 5D). In BMDM-HCC coculture systems, miR-32^CKO^ mice showed significant reductions in the expression of proinflammatory genes, including CD80, CD86, inducible nitric oxide synthase (iNOS), and CCL2 (Fig. 5E). WB exposure confirmed a decline in the expression of the CD86 and CCL2 protein in the miR-32^CKO^ group (Fig. 5F). Immunofluorescence analysis revealed lower levels of CD86 expression and lipid peroxidation in miR-32^CKO^ BMDMs compared to miR-32^f/f^ controls (Fig. 5G and H). In vivo tumor models demonstrated that miR-32^CKO^ mice exhibited increased tumor volume and weight compared to miR-32^f/f^ controls (Fig. 5I to L), with divergent growth patterns becoming apparent by day 17. Body weight did not differ significantly between groups (Fig. 5M). Immunohistochemical analysis revealed increased Ki67^+^ cells in miR-32^CKO^ tumor tissues (Fig. 5N and O) and reduced CD86 and CD8 expression (Fig. 5P and Q), suggesting that miR-32 deletion promotes tumor cell proliferation while suppressing the antitumor activity of macrophages and CD8^+^ T cells.

### miR-32-5p transforms the antitumor immune microenvironment by triggering the cGAS-STING pathway

**Fig. 6.**
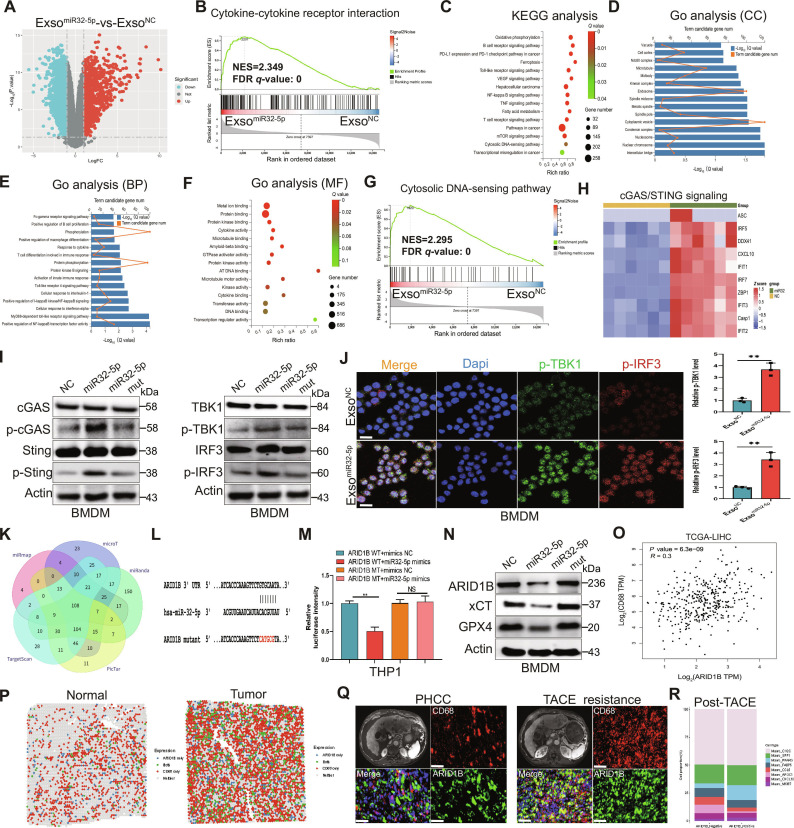
miR-32-5p controls exosomal composition and function via stimulation of the cGAS-STING signaling pathway. (A) Volcano plot presenting differential gene expression in macrophages following miR-32-5p–containing exosome exposure versus NC. (B) GSEA displaying enrichment of cytokine–cytokine receptor interaction pathways. (C) KEGG pathway enrichment for the altered gene expression. (D to F) Gene Ontology (GO) functional annotation demonstrating cellular component (CC), biological process (BP), and molecular function (MF) classifications. (G) GSEA profile of cytosolic DNA-sensing pathway activation. (H) Heatmap visualization of differentially expressed genes in the cGAS/STING signaling pathway. (I) WB analyses of total and phosphorylated cGAS, STING, TBK1, and IRF3 protein levels in BMDMs. (J) Representative immunofluorescence images showing p-TBK1 and p-IRF3 localization in BMDMs. (K) Venn diagram displaying the overlap of predicted miR-32-5p target genes. (L) Predicted miR-32-5p binding sites and target sequence analysis. (M) Dual-luciferase reporter assay showed the luciferase intensity of THP1 cells under different treatment conditions. (N) WB analysis showing ARID1B, xCT, and GPX4 protein expression in BMDMs. (O) Correlation analysis between ARID1B and CD68 expression levels in the TCGA-LIHC cohort. (P) Single-cell spatial transcriptomic profiling compares the spatial distribution of CD68 and ARID1B between normal liver and tumor tissues (data derived from TCGA-LIHC database). (Q) Immunofluorescence microscopy and imaging analyses show ARID1B expression patterns and spatial distribution across PHCC- and TACE-resistant tissues. (R) A hierarchical heatmap displays differential expression profiles of cytokines and signaling molecules within the tumor microenvironment following TACE intervention. Scale bar, 20 μm. *P < 0.05; **P < 0.01; ***P < 0.001.

To characterize the molecular mechanisms underlying miR-32-5p function, we performed transcriptomic analysis comparing macrophages treated with exso^miR-32-5p^ versus exso^NC^ (Fig. 6A). Gene Set Enrichment Analysis (GSEA) revealed significant enrichment of the cytokine–cytokine receptor interaction pathway and, notably, the cytosolic DNA-sensing pathway in exso^miR-32-5p^-treated cells (Fig. 6B and G), suggesting the involvement of immune activation processes. Subsequent pathway analyses further supported a role for miR-32-5p in immune regulation and signal transduction (Fig. 6C to F), with heatmap analysis revealing distinct expression patterns of genes associated with the cyclic GMP-AMP synthase (cGAS)–stimulator of interferon genes (STING) signaling pathway (Fig. 6H). Consistent with these findings, WB analysis demonstrated that exso^miR-32-5p^-treated BMDMs exhibited increased phosphorylation of cGAS and STING, without notable changes in total protein levels, as well as elevated phosphorylation of downstream effectors TBK1 and IRF3 (Fig. 6I). These observations were further corroborated by immunofluorescence microscopy, which confirmed increased phosphorylation of TBK1 and IRF3 (Fig. 6J). The bioinformatics analyses were conducted to determine the possible miR-32-5p target (Fig. 6K and L), and dual-luciferase reporter assay showed that the luciferase intensity of THP1 cells was obviously decreased with miR-32-5p mimic treatment but not in mutant cells with disrupted binding sites (Fig. 6M). SLC7A11 encodes the xCT protein, a cystine–glutamate antiporter that plays a key role in maintaining cellular redox homeostasis through glutathione synthesis. Notably, elevated SLC7A11 expression has been linked to poor prognosis across multiple cancer types, underscoring its importance in cancer progression and therapeutic resistance [24,25]. The analysis of WB proved the differentiation in the expression of ARID1B, XCT, and GPX4 (Fig. 6N). Correlation analysis using the The Cancer Genome Atlas–Liver Hepatocellular Carcinoma (TCGA-LIHC) database revealed a significant positive correlation between ARID1B and CD68 expression (Fig. 6O). Furthermore, ARID1B levels were significantly elevated in HCC tumor tissues compared to normal hepatic tissues, and high ARID1B expression correlated with poor survival in HCC patients according to International Cancer Genome Consortium (ICGC) and TCGA databases (Fig. S2A to C), suggesting ARID1B as a potential therapeutic target. Additional research proved that ferroptosis activation is closely associated with the expression of miR-32-5p (Fig. S3A). ARID1B as a potential diagnostic biomarker in several malignancies has been increasingly recognized, and the inhibition of ARID1B has been reported to activate cGAS-STING [26,27]. Notably, ARID1B expression affects immune cell infiltration patterns in the HCC microenvironment, and miR-32-5p has been identified as a key regulator of cellular responses to oxidative stress and ferroptosis by targeting ARID1B (Fig. S3B and C). This regulation leads to significant changes in immune cell composition within tumor tissue (Fig. 6P). Furthermore, immunofluorescence analysis revealed increased colocalization of ARID1B with the macrophage marker CD68 in TACE-resistant specimens (Fig. 6Q). Heatmap analysis was performed to visualize the expression dynamics of key molecules in the TME following TACE treatment (Fig. 6R). Overall, these findings demonstrate that miR-32-5p enhances antitumor immunity by activating the cGAS-STING pathway through phosphorylation of key signaling molecules while simultaneously suppressing ARID1B expression to relieve its inhibitory effect on cGAS-STING signaling, ultimately contributing to the reversal of TACE resistance in HCC.

### Therapeutic efficacy of exosomal miR-32-5p through ferroptosis induction in HCC

**Fig. 7.**
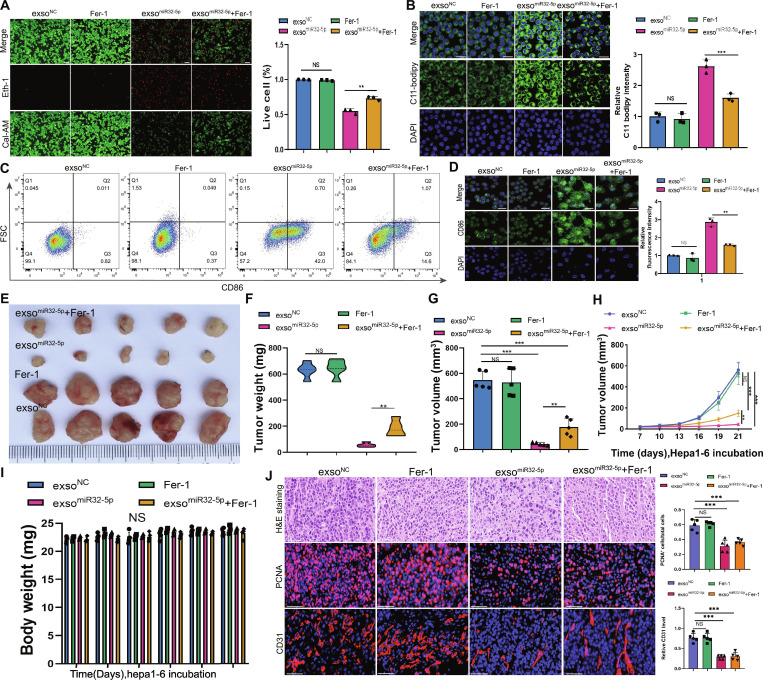
Therapeutic effects of miR-32-5p exosomes combined with ferroptosis inhibitor in HCC. (A) Cell viability assessment of BMDMs across treatment groups by live-dead cell staining. (B) Quantification of lipid peroxidation levels by C11-BODIPY fluorescence intensity analysis. (C) Flow cytometric analysis of CD86 expression. (D) Quantitative analyses of CD86 expression levels. (E) Representative macroscopic images of tumor xenografts. (F) Comparative analysis of tumor weights across treatment groups. (G) Quantitative assessment of tumor volumes among treatment groups. (H) Temporal analysis of tumor volume progression following Hepa1-6 cell inoculation. (I) Longitudinal assessment of mouse body weight across treatment groups, showing no significant intergroup variations. (J) Representative H&E staining and immunohistochemical analysis of PCNA and CD31 in tumor sections. Scale bar, 20 μm. *P < 0.05; **P < 0.01; ***P < 0.001.

To confirm that ferroptosis plays a pivotal role in miR-32-5p-mediated antitumor activity, the ferroptosis inhibitor Fer-1 was employed [28,29]. In vitro experiments demonstrated that exso^miR-32-5p^ treatment significantly affected cell viability and lipid peroxidation, effects that were partially reversed by Fer-1 treatment (Fig. 7A and B). Similarly, Fer-1 partially blocked exso^miR-32-5p^-mediated M1 macrophage polarization (Fig. 7C and D). In vivo, Fer-1 cotreatment partially attenuated the tumor growth inhibition induced by exso^miR-32-5p^ (Fig. 7E to H), confirming ferroptosis as a critical mechanism. The safety profiles of all the treatments were favorable (Fig. 7I). These results were supported by histological studies, which revealed Fer-1 to partially reverse tumor proliferation and angiogenesis inhibited by exso^miR-32-5p^ (Fig. 7J). These observations indicate that miR-32-5p exosomes produce antitumor effects by promoting ferroptosis and polarizing the M1 macrophage, where ferroptosis is a key mechanism.

### Synergistic antitumor effects of combined miR-32-5p exosome and anti-PD-L1 treatment in HCC

**Fig. 8.**
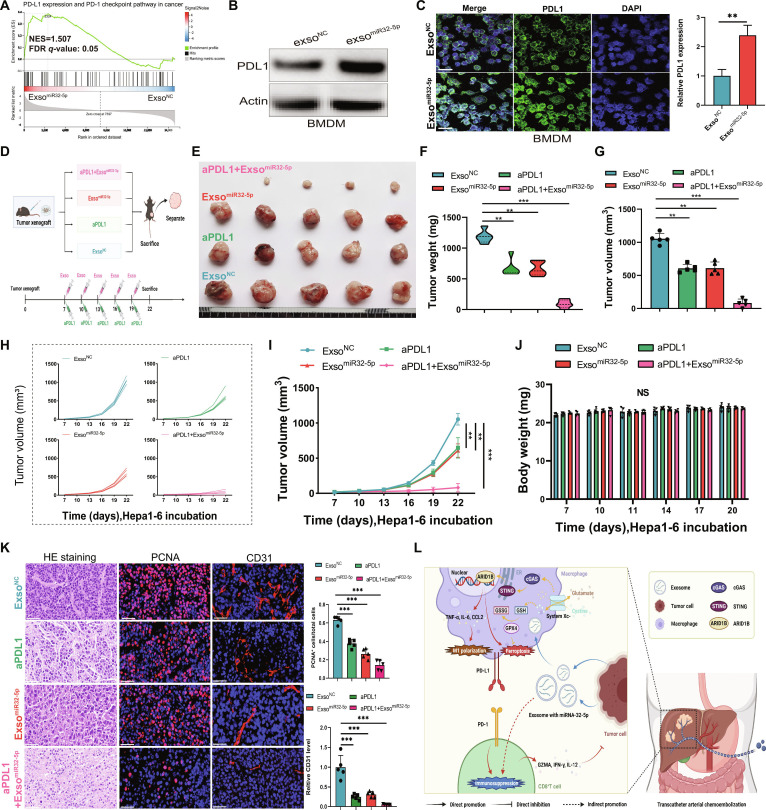
Synergistic antitumor effects of miR-32-5p exosomes combined with PD-L1 inhibition. (A) GSEA profile demonstrating enrichment of PD-L1 signaling pathway genes. (B) WB analysis showing PD-L1 protein levels in BMDMs. (C) Representative immunofluorescence images depicting PD-L1 expression in BMDMs. (D) Schematic diagram depicting the experimental animal system. (E) Macroscopic visualization of tumors from 4 treatment cohorts (exso^NC^, exso^miR-32-5p^, aPD-L1, and exso^miR-32-5p^ + aPD-L1). (F and G) Quantitative analyses of tumor mass and volume across treatment groups. (H and I) Temporal analysis of tumor volume progression. (J) Longitudinal assessment of mouse body weight demonstrating comparable values across treatment groups. (K) Representative images and quantitative analysis of H&E staining, PCNA immunohistochemistry, and CD31 vessel density in tumor sections. (L) Schematic diagram depicting the putative molecular mechanism responsible for the synergistic anticancer effects. Scale bar, 20 μm. *P < 0.05; **P < 0.01; ***P < 0.001.

The high advancements in immunotherapy modalities have emphasized the effective antitumor efficacy of the combination modalities over the monotherapy regimen [30–33]. To evaluate the synergistic antitumor efficacy of miR-32-5p exosomes combined with PD-L1 inhibitors, we performed comprehensive assessments of therapeutic effectiveness and the underlying molecular mechanisms of this combination strategy. GSEA revealed significant enrichment of genes associated with the PD-L1 signaling pathway in exso^miR-32-5p^-treated samples compared to exso^NC^ controls (Fig. 8A). WB and immunofluorescence analysis revealed that miR-32-5p-containing exosomes induced increased PD-L1 protein expression in BMDMs (Fig. 8B and C), and transduction of miR-32-5p into BMDMs also enhanced PD-L1 protein expression (Fig. S4A and B). These observations indicate that miR-32-5p regulates the tumor immune microenvironment through modulation of PD-L1 expression. Building on this finding, we evaluated the therapeutic potential of combining exso^miR-32-5p^ with anti-PD-L1 treatment in a mouse liver cancer model. The combination therapy exhibited superior tumor suppression compared to either agent alone, as evidenced by significantly reduced tumor volume and weight (Fig. 8D to G). Tumor growth curves further demonstrated markedly slower progression in the combination therapy group (Fig. 8H and I). Importantly, body weight remained stable across all treatment groups (Fig. 8J), and no significant alterations were observed in blood biochemistry parameters including albumin (ALB), alanine aminotransferase (ALT), aspartate aminotransferase (AST), blood urea nitrogen (BUN), creatinine (CR), and creatine kinase (CK) (Fig. S5A and B), indicating favorable safety profiles. Histological examination of tumor tissues showed reduced cell density, decreased proportions of PCNA-positive proliferating cells, and fewer CD31-positive vessels in the combination-treated group (Fig. 8K). Collectively, these findings demonstrate that miR-32-5p-enriched exosomes enhance antitumor immunity through PD-L1 regulation and act synergistically with PD-L1 blockade to achieve effective tumor suppression and prolonged survival, establishing a promising combinatorial immunotherapy strategy for liver cancer.

## Discussion

HCC remains one of the leading causes of cancer-related mortality worldwide, with treatment options constrained by tumor heterogeneity and therapeutic resistance [34,35]. Despite advances in surgical resection, transplantation, and systemic therapies, the inherent heterogeneity of HCC continues to drive high recurrence rates and therapeutic resistance [36–38]. TACE is widely used as a first-line treatment for intermediate- and advanced-stage HCC; however, its therapeutic efficacy is frequently compromised by the complex TME and the emergence of resistance mechanisms [39–41]. Recent studies have identified ferroptosis as a promising strategy to overcome therapeutic resistance in cancer [42–45], and have highlighted macrophage reprogramming as a critical factor in tumor development [46,47]. Numerous studies have explored strategies targeting ferroptosis and macrophage reprogramming to inhibit HCC progression [48–51]. Building upon our previous research, this study identifies a novel therapeutic strategy in which exosomal miR-32-5p regulates TAM ferroptosis to inhibit HCC progression and enhance the efficacy of anti-PD-L1 immunotherapy. Our comprehensive analysis revealed that TACE significantly remodels the HCC immune microenvironment, particularly affecting the distribution and activity of CD68^+^ macrophages. Comparative analysis demonstrated notable variations in cellular composition between primary and post-TACE-resistant HCC tissues. Notably, exosomal miR-32-5p expression was significantly decreased in post-TACE-resistant HCC, suggesting its involvement in this microenvironmental remodeling. The negative correlation between miR-32-5p levels and CD68^+^ macrophage density further supports a role for miR-32-5p as a key regulator of macrophage dynamics following TACE treatment. Mechanistically, our experiments demonstrated that exosomal miR-32-5p induces ferroptosis in TAMs through GSH depletion, elevated lipid peroxidation, and increased intracellular iron accumulation, ultimately leading to mitochondrial dysfunction. Importantly, while exso^miR-32-5p^ treatment reduced the viability of protumoral TAMs, it simultaneously promoted macrophage polarization toward an M1 proinflammatory phenotype, as evidenced by increased expression of CD86, CCL2, TNF-α, and IL-6. This dual function—selectively eliminating protumoral macrophages while reprogramming surviving cells toward an antitumor phenotype—represents a promising mechanism for reshaping the tumor immune microenvironment.

To validate the therapeutic potential of these findings, we conducted in vivo experiments demonstrating that exosomal miR-32-5p effectively inhibits tumor growth and metastasis. Treatment with exso^miR-32-5p^ resulted in significant reduction of tumor burden, diminished hepatic metastasis, and decreased expression of proliferation markers. Moreover, exso^miR-32-5p^ stimulated local antitumor immunity by promoting the infiltration of CD86^+^ antigen-presenting cells and CD8^+^ cytotoxic T lymphocytes into the TME, highlighting its dual role in suppressing tumor growth directly while simultaneously enhancing immune responses. Mechanistic investigations revealed that miR-32-5p activates the cGAS-STING pathway by targeting ARID1B, thereby orchestrating tumor immune microenvironment remodeling. GSEA and pathway analyses demonstrated significant enrichment of immune-related signaling cascades in exso^miR-32-5p^-treated samples, with activation of cytosolic DNA-sensing pathways and increased phosphorylation of cGAS, STING, TBK1, and IRF3 further supporting this regulatory axis. ARID1B was validated as a direct target of miR-32-5p, and its down-regulation led to decreased expression of xCT and GPX4, thereby elucidating the molecular link between miR-32-5p, oxidative stress regulation, and ferroptosis induction.

Analysis of combination therapies revealed that exso^miR-32-5p^ exerts antitumor effects through 2 complementary pathways: induction of ferroptosis and stimulation of M1 macrophage activation. The partial reversal of these effects by Fer-1 underscores the critical contribution of ferroptosis to miR-32-5p-mediated tumor suppression. Building on these mechanistic insights, we explored clinically relevant combination strategies and found that exso^miR-32-5p^ combined with PD-L1 blockade produced remarkable synergistic effects. Molecular analysis revealed that exso^miR-32-5p^ treatment up-regulated PD-L1 signaling pathway genes and increased PD-L1 protein levels in BMDMs, providing a rationale for this combination approach. This strategy translated into substantial therapeutic benefits, including enhanced tumor suppression, prolonged survival, and a favorable safety profile. Importantly, comprehensive safety assessments demonstrated high tolerability, with blood biochemistry analyses revealing no significant alterations in liver or kidney function across treatment groups, supporting the translational potential of this combination therapy.

Collectively, these findings advance our understanding of HCC therapy by establishing a novel treatment paradigm: Exosomal miR-32-5p induces ferroptosis in TAMs, thereby suppressing HCC progression and potentiating the efficacy of anti-PD-L1 immunotherapy. This discovery holds significant promise for addressing current challenges in HCC management. Moreover, the identification of exosomal miR-32-5p as a potential biomarker of TACE response may facilitate patient stratification and therapeutic optimization in clinical practice. Notwithstanding these encouraging findings, several limitations merit consideration. While our preclinical models demonstrated substantial therapeutic potential, further validation using patient-derived xenograft models and optimization of dosing regimens are warranted. Additionally, comprehensive long-term toxicology studies in larger animal models will be essential prior to clinical translation.

## Conclusion

This study identified exosomal miR-32-5p as an important modulator of tumor-related macrophage ferroptosis and immune microenvironment reprogramming in HCC. miR-32-5p regulates antitumor immune responses by modulating the ARID1B–cGAS–STING pathway. Its synergy with anti-PD-L1 immunotherapy provides a solid foundation for developing novel combination therapies to improve treatment outcomes in HCC patients.

## Materials and Methods

### Cell culture

The cell line Hepa1-6 of mouse HCC and BMDMs were used in this investigation. Hepa1-6 cells were cultured in full Dulbecco’s modified Eagle’s medium (DMEM) containing 10% fetal bovine serum (FBS) and then incubated in chambers humidly regulated at 37 °C, CO_2_ was controlled at 5%, replacement of the culture medium was also done after a certain period of time, and this ensured maintenance of cell viability. Mice were euthanized by cervical dislocation under isoflurane anesthesia, followed by BMDM isolation. The mice were washed with 75% ethanol solution 5 min. The femur and tibia were accessed using dissection of the posterior limbs, and bone marrow cells were isolated by lavaging serum-free RPMI 1640 medium on the marrow cavity. The cell mixture that was harvested was processed by centrifugation at 1,500 rpm, and the supernatant was removed after 5 min. The resulting cell pellet was exposed to 1 ml of red blood cell lysis buffer for a 5-min period to remove red blood cells. After another centrifugation step and supernatant disposal, bone marrow cells were suspended in RPMI 1640 medium supplemented with 20 ng/ml macrophage colony-stimulating factor (M-CSF) and 10% FBS. Cell culture was maintained at 37 °C in a humidified atmosphere containing 5% CO_2_ for 7 d, with medium changes performed every 3 d. Differentiated BMDMs were obtained for subsequent assays. Standard culture parameters (37 °C, 5% CO_2_) were used for all cell types.

### Animal experiments

The study utilized miR-32 conditional knockout mice (miR-32^CKO^) along with their corresponding wild-type controls (miR-32^f/f^) as experimental subjects. Macrophage-specific deletion of miR-32 was accomplished through the LysM-Cre recombinase system. C57BL/6 mice aged 6 to 8 weeks were used as experimental subjects and were housed under standard specific pathogen-free (SPF) conditions with a 12-h light–dark cycle and unrestricted access to food and water. At study conclusion, animals underwent humane euthanasia via cervical dislocation after isoflurane anesthesia. Each experimental group contained no fewer than 5 mice, and all experiments were conducted in triplicate to guarantee data reliability.

### Exosome isolation and identification

Exosome isolation was performed from human monocytes and cellular culture supernatants. Upon reaching 80% confluence, cells underwent cultivation in serum-free medium for 24 h. Collected supernatant was subjected to low-speed centrifugation to eliminate cellular debris. Exosome separation occurred through differential ultracentrifugation (10,000*g* for 30 min, 100,000*g* for 70 min). Following resuspension of exosome pellets in phosphate-buffered saline (PBS) and subsequent ultracentrifugation, purified exosomes were preserved at −80 °C for later experimental use.

### RNA preparation and real-time quantitative polymerase chain reaction analysis

Total RNA isolation was performed by homogenizing tissue samples in 1 ml of TRIzol reagent using ribonuclease (RNase)-free 1.5-ml tubes. Sample mixing was achieved by tube inversion 10 to 15 times, and samples remained on ice for 5 min. Following 4 °C centrifugation for 5 min, 1,000 μl of supernatant was moved to a fresh tube. Chloroform addition (200 μl) maintained TRIzol:chloroform ratio at 5:1 under reduced light conditions, tube inversion followed (10 to 15 times), ice incubation proceeded for 5 min, and centrifugation at 4 °C lasted 15 min. Aqueous phase (400 to 500 μl) was moved to a fresh tube and combined with equivalent isopropanol volume. The mixture underwent inversion and ice incubation for 10 min. Following centrifugation at 4 °C for 10 min, the supernatant was aspirated, and the RNA pellet underwent washing with 1 ml of 75% ethanol. After centrifugation at 4 °C for 5 min and supernatant aspiration, the pellet was air-dried under aseptic conditions for 10 min before resuspension in RNase-free water. RNA (1 μg) underwent reverse transcription via reverse transcription kit, and the generated cDNA was preserved at −20 °C for later polymerase chain reaction (PCR) analysis.

### WB analysis

Total protein extraction was performed using radioimmunoprecipitation assay (RIPA) lysis buffer containing 1% protease and phosphatase inhibitor cocktail. Following centrifugation at 12,000*g* for 20 min, the supernatants were collected and protein concentrations were determined by bicinchoninic acid (BCA) protein assay. Equal amounts of protein were resolved on 10% to 12% sodium dodecyl sulfate–polyacrylamide gel electrophoresis (SDS-PAGE) and transferred onto polyvinylidene fluoride (PVDF) membranes. The membranes were blocked with 5% nonfat dry milk for 1 h at room temperature. Membranes were incubated overnight using primary antibodies at 4 °C. The study was followed by the incubation of the secondary antibodies, which had horseradish peroxidase (HRP) conjugate. The second step lasted 2 h under room temperature. Protein band visualization was done using an enhanced chemiluminescence (ECL) detecting kit and visualized using a chemiluminescence imaging device. ImageJ software was employed to determine band intensities, and β-actin was utilized as a loading control for normalizing the target protein expression.

### Flow cytometry

In the case of surface marker analysis, cells were washed in PBS and single-cell suspension was prepared. Cell concentration was corrected to 1 × 10^6^ cells/ml. Cells were stained with dead cell exclusion dye and fluorophore-conjugated antibodies targeting CD86 and CD206, followed by incubation in the dark at 4 °C for 30 min and subsequent washing. In order to assess lipid peroxidation, cellular labeling with C11-BODIPY staining was done. Flow cytometry was performed on the minimum viable of 30,000 live cells per sample, and 3 experiments were repetitive. Data analysis was done using FlowJo software. The positive cell percentages, mean fluorescence intensity, and intergroup differences were statistically determined under analysis of variance (ANOVA) or *t* tests, and *P* < 0.05 was considered as statistically significant.

### Immunofluorescence and imaging

Cell proliferation and angiogenesis markers underwent immunofluorescence evaluation. Cells received culture on coverslips, 4% paraformaldehyde fixation occurred post-treatment, 0.3% Triton X-100 permeabilization followed, and 5% BSA blocking was applied. Cells were exposed to primary antibody (PCNA) overnight at 4 °C. The next day, fluorescent secondary antibody was applied to samples for 1 h at ambient temperature. Following DAPI nuclear staining, slides were mounted for observation. Viability of cells was assessed via concurrent staining with Calcein-AM (marking viable cells, green) and EthD-1 (marking nonviable cells, red) at 37 °C for 30 min. Image acquisition occurred via confocal laser scanning microscope, and multiple random fields were captured under uniform settings. Fluorescence intensity and morphological parameters received quantification through ImageJ. Each experiment underwent triplicate performance, and no fewer than 5 independent fields received analysis per condition.

### Cell viability assay

BMDMs were seeded at a density of 5 × 10^3^ cells per well in 96-well plates and cultured in 100 μl of complete medium supplemented with 10% FBS and 1% penicillin–streptomycin. The cells were incubated at 37 °C in a humidified atmosphere containing 5% CO₂ for 24 h to allow adequate cell attachment. After the designated 24-h treatment period, 10 μl of CCK-8 solution was added to each well, and plates were incubated in the dark under standard culture conditions for 1.5 h. Absorbance measurement occurred at 450 nm via microplate reader. Each experimental condition underwent triplicate testing, and experiments received independent repetition 3 times to guarantee data reproducibility. Data analysis proceeded through GraphPad Prism software, and cell viability calculation represented absorbance percentage in treated groups compared to untreated control groups. The assessment of intergroup statistical differences was conducted through proper analytical tools, and the levels of significance were set to *P* < 0.05. Special care was paid to the uniform distribution of cells in all the wells in order to minimize interwell variability, and the cell environmental culture parameters were constantly monitored to maintain and sustain the stable, ideal conditions throughout the full length of the experiment.

### Transmission electron microscopy

BMDMs cocultured with Hepa1-6 cells were collected, washed with prewarmed PBS (37 °C), separated with 0.25% trypsin, and collected by centrifugation at 1,000*g*. Cell pellets were fixed with 2.5% glutaraldehyde for 2 h, embedded in agarose, and sequentially dehydrated through an ethanol gradient. After replacement with isoamyl acetate, samples were embedded in Epon 812 resin and cured at 60 °C for 48 h. Ultrathin sections (60 to 80 nm) were generated and stained with both uranyl acetate and lead citrate. Exosome internalization and ferroptosis-associated ultrastructural alterations were examined by TEM.

### Tumor model

A xenograft tumor model was established through subcutaneous injection of 1 × 10^6^ Hepa1-6 cells into the right flank of C57BL/6 mice. Tumor dimensions were measured every 3 d using digital calipers, and tumor volume (TV) was calculated using the following formula: TV (mm^3^) = length × width^2^ × 0.5. When tumors reached 50 to 100 mm^3^ in volume, mice were randomly assigned to control, exosome treatment (exso^NC^ and exso^miR-32-5p^, delivered through tail vein injection 3 times weekly), PD-L1 inhibitor (delivered through intraperitoneal injection every 3 to 4 d at 10 mg/kg), and combination treatment groups. Exosome administration 3 times weekly maintains stable therapeutic concentrations based on the pharmacokinetic characteristics of exosomes. Anti-PD-L1 antibody administration every 3 to 4 d follows the standard dosing regimen widely used in preclinical studies, and given the relatively long half-life of antibody therapeutics, this dosing frequency is sufficient to maintain effective therapeutic concentrations and sustained immune checkpoint blockade. Treatment continued for 3 weeks, with systematic monitoring of tumor volume and body weight conducted during this period. At study termination, animals were euthanized, tumor masses were measured, and tissue specimens were collected for further analysis. At least 8 mice per group were used to ensure statistical validity.

### HCC lung metastasis mouse model

An experimental lung metastasis model of HCC was established in 6- to 8-week-old C57BL/6 mice through intravenous injection of 2 × 10^6^ Hepa1-6 cells in 100-μl suspension. Therapeutic intervention was initiated on day 2 post-injection and continued for 3 weeks. Metastatic progression was monitored via in vivo bioluminescence imaging throughout the study duration. At experimental conclusion, mice were euthanized and pulmonary tissues were harvested to evaluate surface metastatic foci and determine the lung-to-body weight ratio. Metastatic burden was evaluated by quantifying pulmonary nodules and performing dimensional analysis using hematoxylin and eosin (H&E) staining and immunohistochemistry. No fewer than 8 mice per group were employed to ensure reliable results.

### Histological analysis

Tissue specimens received immediate 4% paraformaldehyde fixation for 24 to 48 h, dehydration through graded ethanol series followed, and xylene clearance and paraffin embedding proceeded. Serial 4-μm sections underwent microtome preparation, and histomorphological analysis occurred through H&E staining. Sections received deparaffinization and rehydration, hematoxylin staining proceeded for 5 min, running water washing followed, acid alcohol differentiation occurred, and running water bluing was performed. Eosin counterstaining lasted 2 min, and sequential dehydration, clearing, and mounting followed. Histological evaluation received performance by experienced pathologists.

### Gene Ontology enrichment analysis and Kyoto Encyclopedia of Genes and Genomes pathway analysis

Differential expression analysis was performed using the limma R package, and Kyoto Encyclopedia of Genes and Genomes (KEGG) pathway enrichment was conducted through the Dr.Tom platform (https://biosys.bgi.com/) to identify signaling pathways associated with differentially expressed genes. Statistical significance was defined as an adjusted *P* value of <0.05. All analyses were independently repeated no fewer than 3 times to ensure reproducibility.

### Statistical analysis

Statistical analyses were performed using GraphPad Prism software. Results are reported as mean ± SEM. Between-group comparisons for 2 groups utilized 2-tailed Student’s *t* tests, whereas multiple group analyses employed one-way ANOVA followed by Tukey’s post hoc testing. Data that did not follow normal distribution were analyzed using appropriate nonparametric methods. Survival analysis was conducted using the Kaplan–Meier approach and assessed by the log-rank test. Correlation analyses employed Pearson or Spearman correlation coefficients based on data characteristics. A *P* value of <0.05 denoted statistical significance, represented as follows: ns (not significant), *P < 0.05, **P < 0.01, ***P < 0.001, ****P < 0.0001. Each experiment was repeated independently a minimum of 3 times with proper controls to validate reliability and reproducibility.

## Data Availability

All data generated or analyzed during this study are included in this current article and its additional files.

## References

[1] Chen L, Wei X, Gu D, Xu Y, Zhou H. Human liver cancer organoids: Biological applications, current challenges, and prospects in hepatoma therapy. Cancer Lett. 2023;555: Article 216048. 36603689 10.1016/j.canlet.2022.216048

[2] El-Serag HB, Rudolph KL. Hepatocellular carcinoma: Epidemiology and molecular carcinogenesis. Gastroenterology. 2007;132(7):2557–2576. 17570226 10.1053/j.gastro.2007.04.061

[3] Su XZ, Qiao EQ, Teng GJ, Xiong F. Quaternary ammonium salt microspheres loaded with vascular disrupting agents for targeted interventional therapy of hepatocellular carcinoma. Acta Biomater. 2025;203:591–603. 40716478 10.1016/j.actbio.2025.07.055

[4] Gong F, Zheng L, Xu J, Wu Y, Jin Q, Lu J, Pei Z, Zhao Z, Chen M, Tu J, et al. Magnesium microspheres for enhanced transarterial chemoembolization therapy of hepatocellular carcinoma: From animal models to a pilot clinical study. Sci Adv. 2025;11(27): Article eadv0885. 40601735 10.1126/sciadv.adv0885 PMC12219493

[5] Lang D, Agarwal R, Brown SA, Borgmann AJ, Lockney NA, Goff LW, Heumann TR. Multidisciplinary care and multimodal treatment approaches for unresectable hepatocellular carcinoma. Adv Oncol. 2024;4(1):247–262. 38882260 10.1016/j.yao.2024.02.002 PMC11178262

[6] Tang D, Chen X, Kang R, Kroemer G. Ferroptosis: Molecular mechanisms and health implications. Cell Res. 2021;31(2):107–125. 33268902 10.1038/s41422-020-00441-1 PMC8026611

[7] Li J, Cao F, Yin HL, Huang ZJ, Lin ZT, Mao N, Sun B, Wang G. Ferroptosis: Past, present and future. Cell Death Dis. 2020;11(2):88. 32015325 10.1038/s41419-020-2298-2 PMC6997353

[8] Yuan H, Pratte J, Giardina C. Ferroptosis and its potential as a therapeutic target. Biochem Pharmacol. 2021;186: Article 114486. 33631189 10.1016/j.bcp.2021.114486

[9] Tang B, Zhu J, Wang Y, Chen W, Fang S, Mao W, Xu Z, Yang Y, Weng Q, Zhao Z, et al. Targeted xCT-mediated ferroptosis and protumoral polarization of macrophages is effective against HCC and enhances the efficacy of the anti-PD-1/L1 response. Adv Sci. 2023;10(2): Article e2203973. 10.1002/advs.202203973 PMC9839855 36442849

[10] Dixon SJ. Epigenetic regulation of ferroptosis in the liver. Research. 2024;7:0323. 38384329 10.34133/research.0323 PMC10880165

[11] Zhang X, Tang B, Luo J, Yang Y, Weng Q, Fang S, Zhao Z, Tu J, Chen M, Ji J. Cuproptosis, ferroptosis and PANoptosis in tumor immune microenvironment remodeling and immunotherapy: Culprits or new hope. Mol Cancer. 2024;23(1):255. 39543600 10.1186/s12943-024-02130-8 PMC11566504

[12] Luo J, Xu L, Feng J, Xu K, Tian P, Bai X, Xu S, Wen L, Lu C, Song J. Tumor microenvironment-activated and ROS-augmented nanoplatform amplified PDT against colorectal cancer through impairing GPX4 to induce ferroptosis. ACS Appl Mater Interfaces. 2025;17(29):41586–41596. 40629874 10.1021/acsami.5c05523

[13] Zhang M, Wang Q, Li C, Chen M, Wang C, Wang Z, Xia T, Yi C, Shi S. Ion interference induced by Ca-Mn nanoplatform enhances ferroptosis and promotes immune response for osteosarcoma treatment. J Adv Res. 2025. 10.1016/j.jare.2025.06.022 PMC12958199 40518113

[14] Hu J, Cui L, Hou B, Ding X, Liu H, Sun W, Mi Y, Chen Y, Zou Z. Ferroptosis in tumor associated immune cells: A double-edged sword against tumors. Crit Rev Oncol Hematol. 2025;212: Article 104818. 40570995 10.1016/j.critrevonc.2025.104818

[15] Li X, Møller SH, Park J, Chuang YM, Hsueh PC, Chang TH, Kao KC, Gallart-Ayala H, Wang YH, Peng JJ, et al. Tumor-instructed glutamine synthesis in cancer-associated fibroblasts promotes pro-tumor macrophages. J Exp Med. 2025;222(9): Article e20241426. 40668214 10.1084/jem.20241426

[16] Yang Y, Li S, To KKW, Zhu S, Wang F, Fu L. Tumor-associated macrophages remodel the suppressive tumor immune microenvironment and targeted therapy for immunotherapy. J Exp Clin Cancer Res. 2025;44(1):145. 40380196 10.1186/s13046-025-03377-9 PMC12083052

[17] Li Y, You J, Zou Z, Sun G, Shi Y, Sun Y, Xu S, Zhang X. Decoding the tumor microenvironment: Exosome-mediated macrophage polarization and therapeutic frontiers. Int J Biol Sci. 2025;21(9):4187–4214. 40612677 10.7150/ijbs.114222 PMC12223767

[18] Mashouri L, Yousefi H, Aref AR, Ahadi AM, Molaei F, Alahari SK. Exosomes: Composition, biogenesis, and mechanisms in cancer metastasis and drug resistance. Mol Cancer. 2019;18(1):75. 30940145 10.1186/s12943-019-0991-5 PMC6444571

[19] Azmi AS, Bao B, Sarkar FH. Exosomes in cancer development, metastasis, and drug resistance: A comprehensive review. Cancer Metastasis Rev. 2013;32(3-4):623–642. 23709120 10.1007/s10555-013-9441-9 PMC3843988

[20] Wang G, Yang Q, Han Y, Zhang Y, Pan W, Ma Z, Tian H, Qu X. miR-32-5p suppresses the progression of hepatocellular carcinoma by regulating the GSK3β/NF-κB signaling. Acta Biochim Biophys Sin. 2025;57(7):1125–1138. 40170617 10.3724/abbs.2025038 PMC12368002

[21] Yuan P, Tang C, Chen B, Lei P, Song J, Xin G, Wang Z, Hui Y, Yao W, Wang G, et al. miR-32-5p suppresses the proliferation and migration of pancreatic adenocarcinoma cells by targeting TLDC1. Mol Med Rep. 2021;24(5):752. 34468015 10.3892/mmr.2021.12392 PMC8430301

[22] Wu R, Guo W, Qiu X, Wang S, Sui C, Lian Q, Wu J, Shan Y, Yang Z, Yang S, et al. Comprehensive analysis of spatial architecture in primary liver cancer. Sci Adv. 2021;7(51): Article eabg3750. 34919432 10.1126/sciadv.abg3750 PMC8683021

[23] Tan J, Fan W, Liu T, Zhu B, Liu Y, Wang S, Wu J, Liu J, Zou F, Wei J, et al. TREM2+ macrophages suppress CD8+ T-cell infiltration after transarterial chemoembolisation in hepatocellular carcinoma. J Hepatol. 2023;79(1):126–140. 36889359 10.1016/j.jhep.2023.02.032

[24] Jin Y, Cai S, Zhou Y, Guo D, Zeng Y, Xu W, Sun Y, Shi Y, Xu Z, Liu Z, et al. Targeting SLC7A11/xCT improves radiofrequency ablation efficacy of HCC by dendritic cells mediated anti-tumor immune response. iMeta. 2024;3(6): Article e248. 39742309 10.1002/imt2.248 PMC11683471

[25] Guo D, Cai S, Deng L, Xu W, Fu S, Lin Y, Jiang T, Li Q, Shen Z, Zhang J, et al. Ferroptosis in pulmonary disease and lung cancer: Molecular mechanisms, crosstalk regulation, and therapeutic strategies. MedComm. 2025;6(3): Article e70116. 39991627 10.1002/mco2.70116 PMC11847630

[26] Zhu Y, Yan C, Wang X, Xu Z, Lv J, Xu X, Yu W, Zhou M, Yue L. Pan-cancer analysis of ARID family members as novel biomarkers for immune checkpoint inhibitor therapy. Cancer Biol Ther. 2022;23(1):104–111. 35239432 10.1080/15384047.2021.2011643 PMC8896200

[27] Zhu G, Liu J, Li Y, Huang H, Chen C, Wu D, Cao P, Su L, Wang Y, Zhang H, et al. ARID1B deficiency leads to impaired DNA damage response and activated cGAS-STING pathway in non-small cell lung cancer. J Cancer. 2024;15(9):2601–2612. 38577613 10.7150/jca.91955 PMC10988295

[28] Miotto G, Rossetto M, Di Paolo ML, Orian L, Venerando R, Roveri A, Vuvkovic A-M, Travain VB, Zaccarin M, Zennaro L, et al. Insight into the mechanism of ferroptosis inhibition by ferrostatin-1. Redox Biol. 2020;28: Article 101328. 31574461 10.1016/j.redox.2019.101328 PMC6812032

[29] Skouta R, Dixon SJ, Wang J, Dunn DE, Orman M, Shimada K, Rosenberg PA, Lo DC, Weinberg JM, Linkermann A, et al. Ferrostatins inhibit oxidative lipid damage and cell death in diverse disease models. J Am Chem Soc. 2014;136(12):4551–4556. 24592866 10.1021/ja411006a PMC3985476

[30] Lee D, Cho M, Kim E, Seo Y, Cha JH. PD-L1: From cancer immunotherapy to therapeutic implications in multiple disorders. Mol Ther. 2024;32(12):4235–4255. 39342430 10.1016/j.ymthe.2024.09.026 PMC11638837

[31] Liu X, He J, Ying H, Chen C, Zheng C, Luo P, Zhu W, Wei T, Tang B, Zhang J. Targeting PFKFB4 biomimetic codelivery system synergistically enhances ferroptosis to suppress small cell lung cancer and augments the efficacy of anti-PD-L1 immunotherapy. Adv Sci. 2025;12(22): Article e2417374. 10.1002/advs.202417374 PMC12165097 40213972

[32] Dermani FK, Samadi P, Rahmani G, Kohlan AK, Najafi R. PD-1/PD-L1 immune checkpoint: Potential target for cancer therapy. J Cell Physiol. 2019;234(2):1313–1325. 30191996 10.1002/jcp.27172

[33] Wu Y, Chen C, Xu ZP, Gu W. PD-L1 distribution and perspective for cancer immunotherapy-blockade, knockdown, or inhibition. Front Immunol. 2019;10: Article 2022. 31507611 10.3389/fimmu.2019.02022 PMC6718566

[34] Wang Y, Deng B. Hepatocellular carcinoma: Molecular mechanism, targeted therapy, and biomarkers. Cancer Metastasis Rev. 2023;42(3):629–652. 36729264 10.1007/s10555-023-10084-4

[35] Koshy A. Evolving global etiology of hepatocellular carcinoma (HCC): Insights and trends for 2024. J Clin Exp Hepatol. 2025;15(1): Article 102406. 39346785 10.1016/j.jceh.2024.102406 PMC11426038

[36] de Angelis N, Landi F, Carra MC, Azoulay D. Managements of recurrent hepatocellular carcinoma after liver transplantation: A systematic review. World J Gastroenterol. 2015;21(39):11185–11198. 26494973 10.3748/wjg.v21.i39.11185 PMC4607916

[37] Kulik L, Abecassis M. Living donor liver transplantation for hepatocellular carcinoma. Gastroenterology. 2004;127(5 Suppl 1):S277–S282. 15508095 10.1053/j.gastro.2004.09.042

[38] Tang Z, Zhou X, Lin Z, Yang B, Ma Z, Ye S, Wu Z, Fan J, Liu Y, Liu K, et al. Surgical treatment of hepatocellular carcinoma and related basic research with special reference to recurrence and metastasis. Chin Med J. 1999;112(10):887–891. 11717970

[39] Pleguezuelo M, Marelli L, Misseri M, Germani G, Calvaruso V, Xiruochakis E, Manousou P, Burroughs AK. TACE versus TAE as therapy for hepatocellular carcinoma. Expert Rev Anticancer Ther. 2008;8(10):1623–1641. 18925854 10.1586/14737140.8.10.1623

[40] Chang Y, Jeong SW, Young Jang J, Jae Y. Recent updates of transarterial chemoembolilzation in hepatocellular carcinoma. Int J Mol Sci. 2020;21(21):8165. 33142892 10.3390/ijms21218165 PMC7662786

[41] Zhong BY, Jin ZC, Chen JJ, Zhu HD, Zhu XL. Role of transarterial chemoembolization in the treatment of hepatocellular carcinoma. J Clin Transl Hepatol. 2023;11(2):480–489. 36643046 10.14218/JCTH.2022.00293 PMC9817054

[42] Zheng Y, Wang Y, Lu Z, Wan J, Jiang L, Song D, Wei C, Gao C, Shi G, Zhou J, et al. PGAM1 inhibition promotes HCC ferroptosis and synergizes with anti-PD-1 immunotherapy. Adv Sci. 2023;10(29): Article e2301928. 10.1002/advs.202301928 PMC10582428 37705495

[43] Yao F, Deng Y, Zhao Y, Mei Y, Zhang Y, Liu X, Martinez C, Su X, Rosato RR, Teng H, et al. A targetable LIFR-NF-κB-LCN2 axis controls liver tumorigenesis and vulnerability to ferroptosis. Nat Commun. 2021;12(1):7333. 34921145 10.1038/s41467-021-27452-9 PMC8683481

[44] Zhang D, Man D, Lu J, Jiang Y, Ding B, Su R, Tong R, Chen J, Yang B, Zheng S, et al. Mitochondrial TSPO promotes hepatocellular carcinoma progression through ferroptosis inhibition and immune evasion. Adv Sci. 2023;10(15): Article e2206669. 10.1002/advs.202206669 PMC10214260 36994647

[45] Li Q, Cheng Y, Yang C, Tian M, Wang X, Li D, Li X, Qu J, Zhou S, Zheng L, et al. Targeting the exonic circular OGT RNA/O-GlcNAc transferase/forkhead box C1 axis inhibits asparagine- and alanine-mediated ferroptosis repression in neuroblastoma progression. Research. 2025;8:0703. 40416363 10.34133/research.0703 PMC12099056

[46] Huang J, Pan H, Sun J, Wu J, Xuan Q, Wang J, Ke S, Lu S, Li Z, Feng Z, et al. TMEM147 aggravates the progression of HCC by modulating cholesterol homeostasis, suppressing ferroptosis, and promoting the M2 polarization of tumor-associated macrophages. J Exp Clin Cancer Res. 2023;42(1):286. 37891677 10.1186/s13046-023-02865-0 PMC10612308

[47] Hao X, Zheng Z, Liu H, Zhang Y, Kang J, Kong X, Rong D, Sun G, Sun G, Liu L, et al. Inhibition of APOC1 promotes the transformation of M2 into M1 macrophages via the ferroptosis pathway and enhances anti-PD1 immunotherapy in hepatocellular carcinoma based on single-cell RNA sequencing. Redox Biol. 2022;56: Article 102463. 36108528 10.1016/j.redox.2022.102463 PMC9482117

[48] Tang BF, Xu WT, Fang SJ, Zhu JY, Qiu RF, Shen L, Yang Y, Weng QY, Wang YJ, Ding JY, et al. MELK prevents radiofrequency ablation-induced immunogenic cell death and antitumor immune response by stabilizing FABP5 in hepatocellular malignancies. Mil Med Res. 2025;12(1):5. 39871325 10.1186/s40779-024-00588-7 PMC11773770

[49] Tang B, Zhu J, Shi Y, Wang Y, Zhang X, Chen B, Fang S, Yang Y, Zheng L, Qiu R, et al. Tumor cell-intrinsic MELK enhanced CCL2-dependent immunosuppression to exacerbate hepatocarcinogenesis and confer resistance of HCC to radiotherapy. Mol Cancer. 2024;23(1):137. 38970074 10.1186/s12943-024-02049-0 PMC11225310

[50] Wang L, Zhu L, Liang C, Huang X, Liu Z, Huo J, Zhang Y, Zhang Y, Chen L, Xu H, et al. Targeting N6-methyladenosine reader YTHDF1 with siRNA boosts antitumor immunity in NASH-HCC by inhibiting EZH2-IL-6 axis. J Hepatol. 2023;79(5):1185–1200. 37459919 10.1016/j.jhep.2023.06.021

[51] Zhang J, Tang K, Yang Y, Yang D, Fan W. Advanced nanoprobe strategies for imaging macrophage polarization in cancer immunology. Research. 2025;8:0622. 39990770 10.34133/research.0622 PMC11842672

