# Supplementary material for: Transarterial Chemoembolization Modulates the Exosomal miR-32-5p/cGAS-STING Axis Mediated Macrophage Ferroptosis, Triggers Immune Remodeling, and Enhances Anti-PD-1/L1 Efficacy in HCC
Source: Research (Wash D C). 2026 Jan 27;9:1096. doi: 10.34133/research.1096 (PMC12835496; doi:10.34133/research.1096)
Supplement: Supplementary 1 — Figs. S1 to S5 [file research.1096.f1.zip › Supplementary caption.docx]

**Figure S1. Single-cell transcriptomic landscape analysis revealing distinct cell population signatures in the liver cancer microenvironment**

1. UMAP visualization demonstrating spatial distribution patterns of diverse cellular populations. (B) Hierarchical heatmap illustrating differential expression profiles of signature genes across distinct cell populations.

**Figure S2. Clinical significance of ARID1B expression in HCC**

1. Comparative analysis of ARID1B expression between normal liver and HCC tumor tissues in the TCGA-LIHC cohort, revealing significantly elevated ARID1B levels in tumors. (B) Paired comparison of ARID1B expression in matched normal and tumor tissues from identical patients in the TCGA-LIHC cohort, confirming substantial ARID1B upregulation in malignant samples. (C) Kaplan-Meier survival analysis of TCGA-LIHC HCC patients stratified by ARID1B expression, showing that elevated ARID1B correlates with significantly reduced overall survival.

**Figure S3. Comprehensive analysis of ferroptosis pathway enrichment, immune cell heterogeneity, and ARID1B expression correlations**

(A) GSEA revealing significant ferroptosis pathway activation in exso^miR-32-5p^ samples compared to exso^NC^ controls. (B) Quantitative distribution of macrophage subsets in ALL and PHCC cohorts stratified by ARID1B expression levels. (C) Hierarchical cell type clustering analysis and quantitative distribution of CD8+ T cell subsets under ALL, PHCC, and TACE intervention conditions.

**Figure S4. Enhanced PD-L1 expression in BMDMs following miR-32-5p upregulation**

1. WB analysis revealing enhanced PD-L1 protein levels in mouse BMDMs transfected with miR-32-5p versus miR-NC. (B) Representative immunofluorescence images presenting PD-L1 expression in BMDMs exposed to miR-32-5p compared to miR-NC controls.

**Figure S5. Safety profile assessment of combination therapy with miR-32-5p and anti-PD-L1**

(A-B) Comprehensive blood biochemistry analysis measuring key hepatic and renal function markers: alanine aminotransferase (ALT), aspartate aminotransferase (AST), albumin (ALB), creatine kinase (CK), creatinine (CR), and blood urea nitrogen (BUN).
