# Supplementary figures and images for: Transarterial Chemoembolization Modulates the Exosomal miR-32-5p/cGAS-STING Axis Mediated Macrophage Ferroptosis, Triggers Immune Remodeling, and Enhances Anti-PD-1/L1 Efficacy in HCC
Source: Research (Wash D C). 2026 Jan 27;9:1096. doi: 10.34133/research.1096 (PMC12835496; doi:10.34133/research.1096)

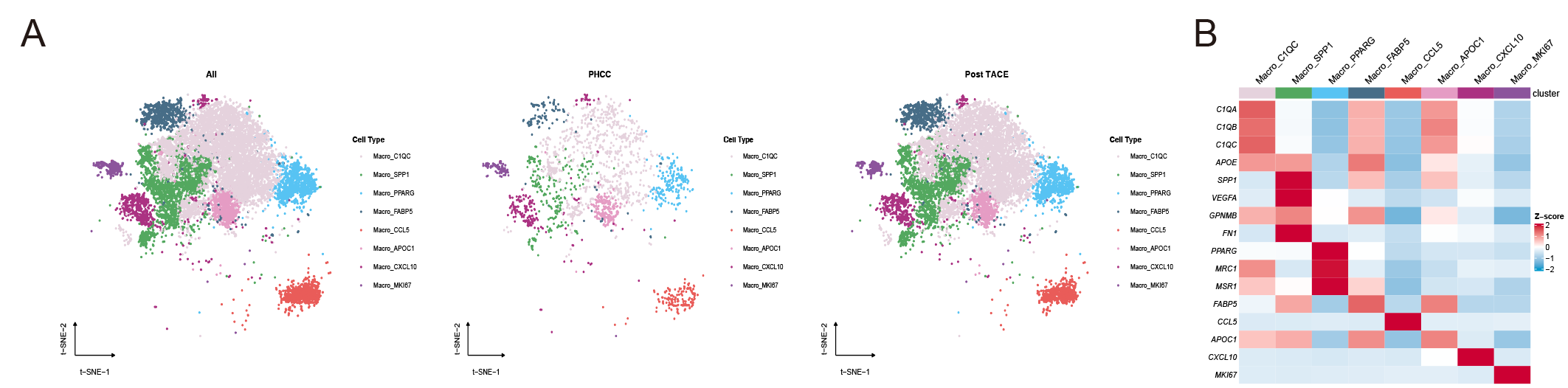

Supplement: Supplementary 1 — Figs. S1 to S5 [file research.1096.f1.zip › figureS1.tif]

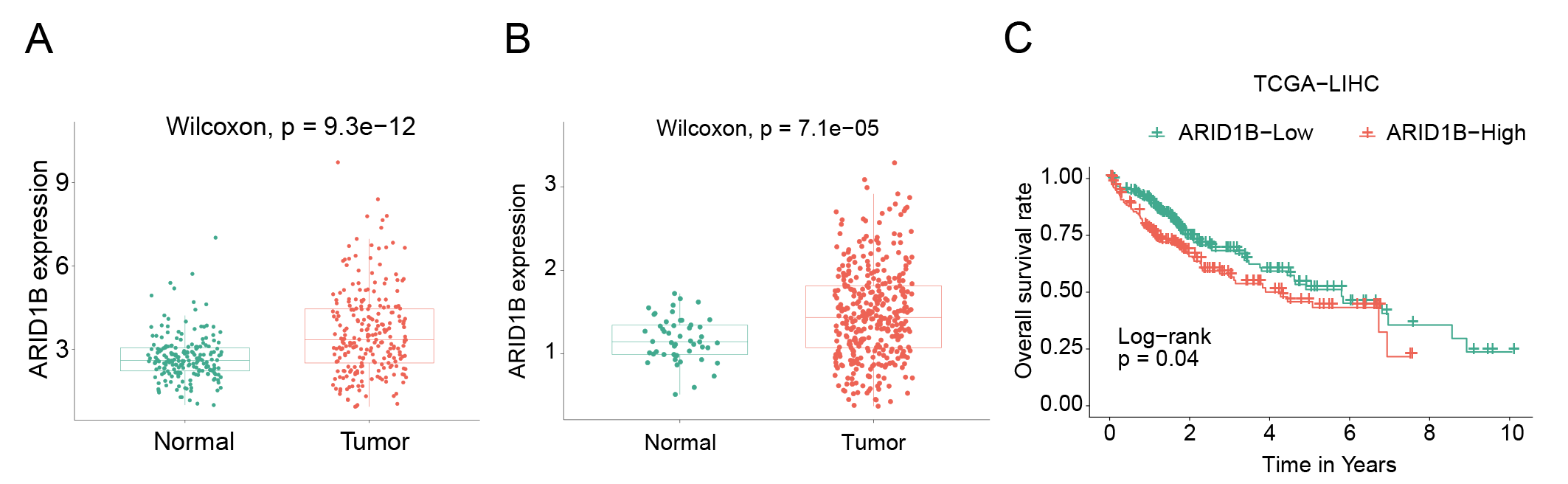

Supplement: Supplementary 1 — Figs. S1 to S5 [file research.1096.f1.zip › FigureS2.tif]

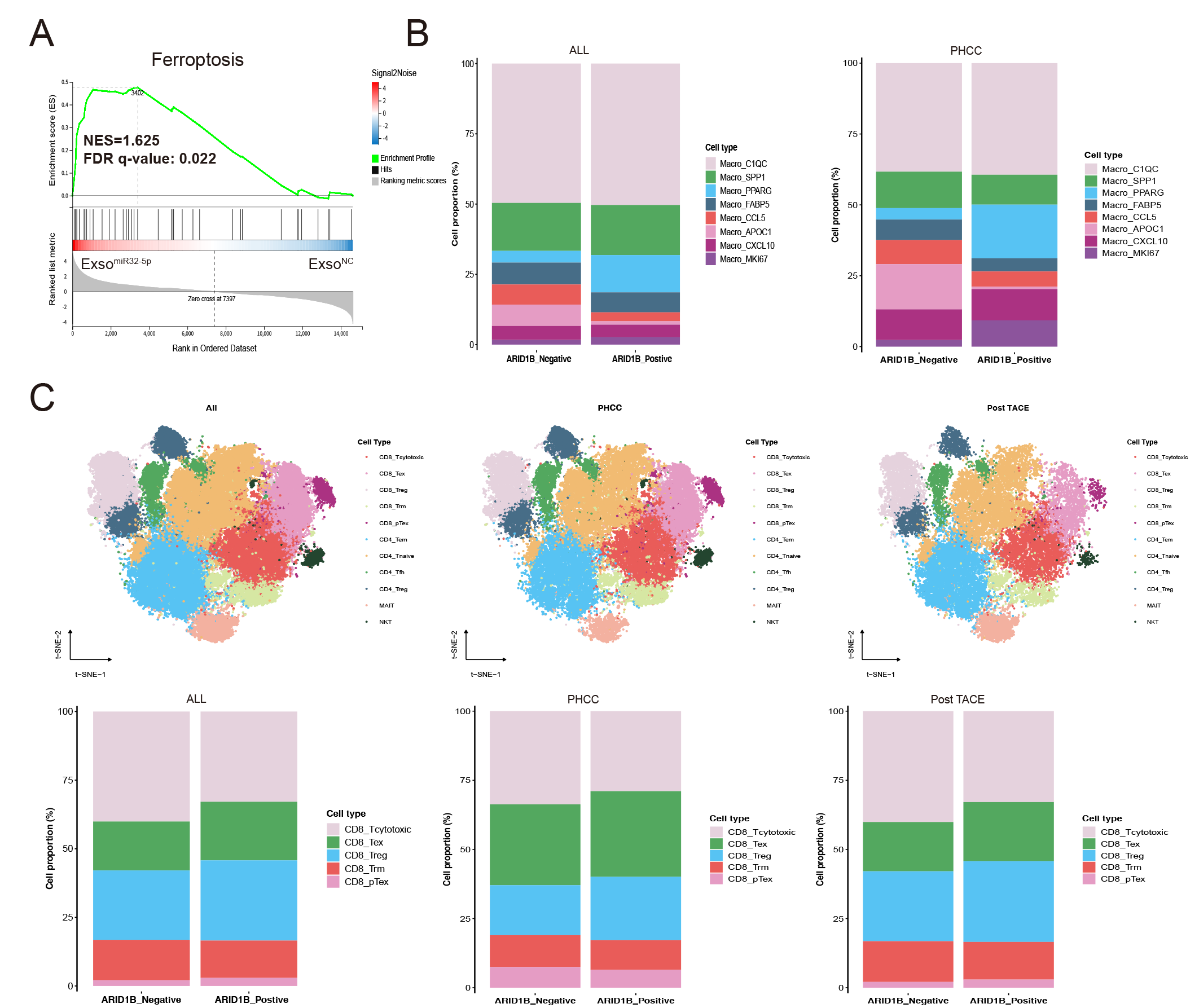

Supplement: Supplementary 1 — Figs. S1 to S5 [file research.1096.f1.zip › figureS3.tif]

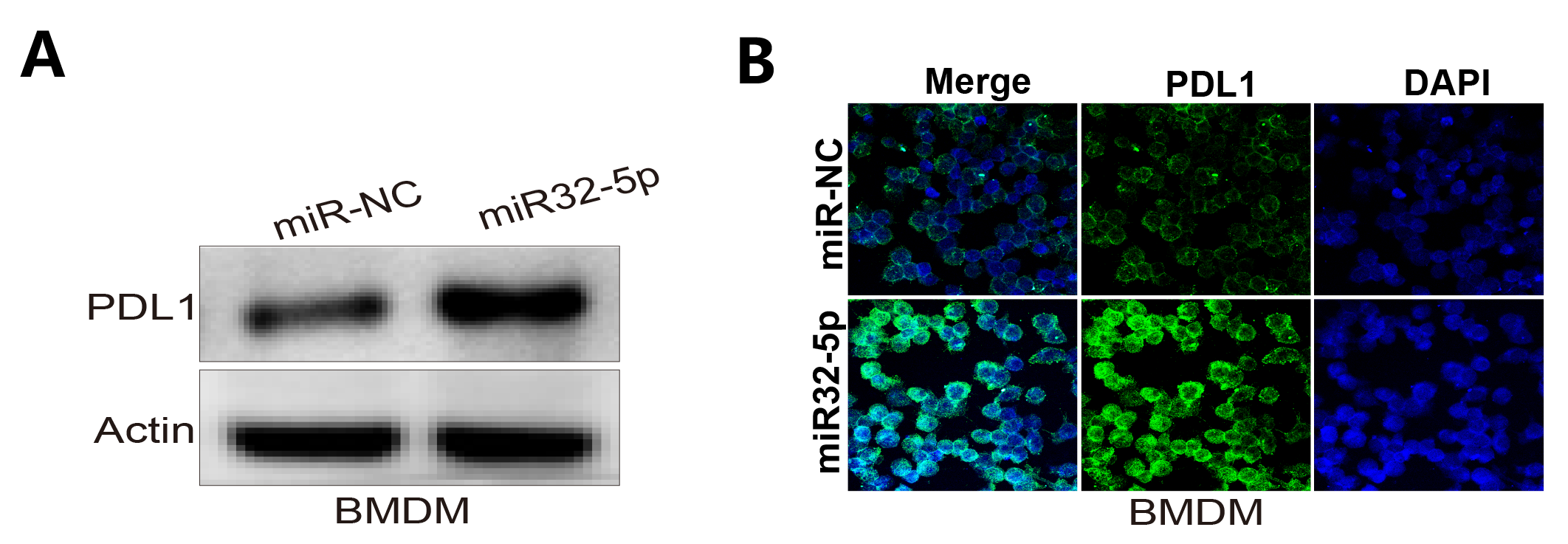

Supplement: Supplementary 1 — Figs. S1 to S5 [file research.1096.f1.zip › FigureS4.tif]

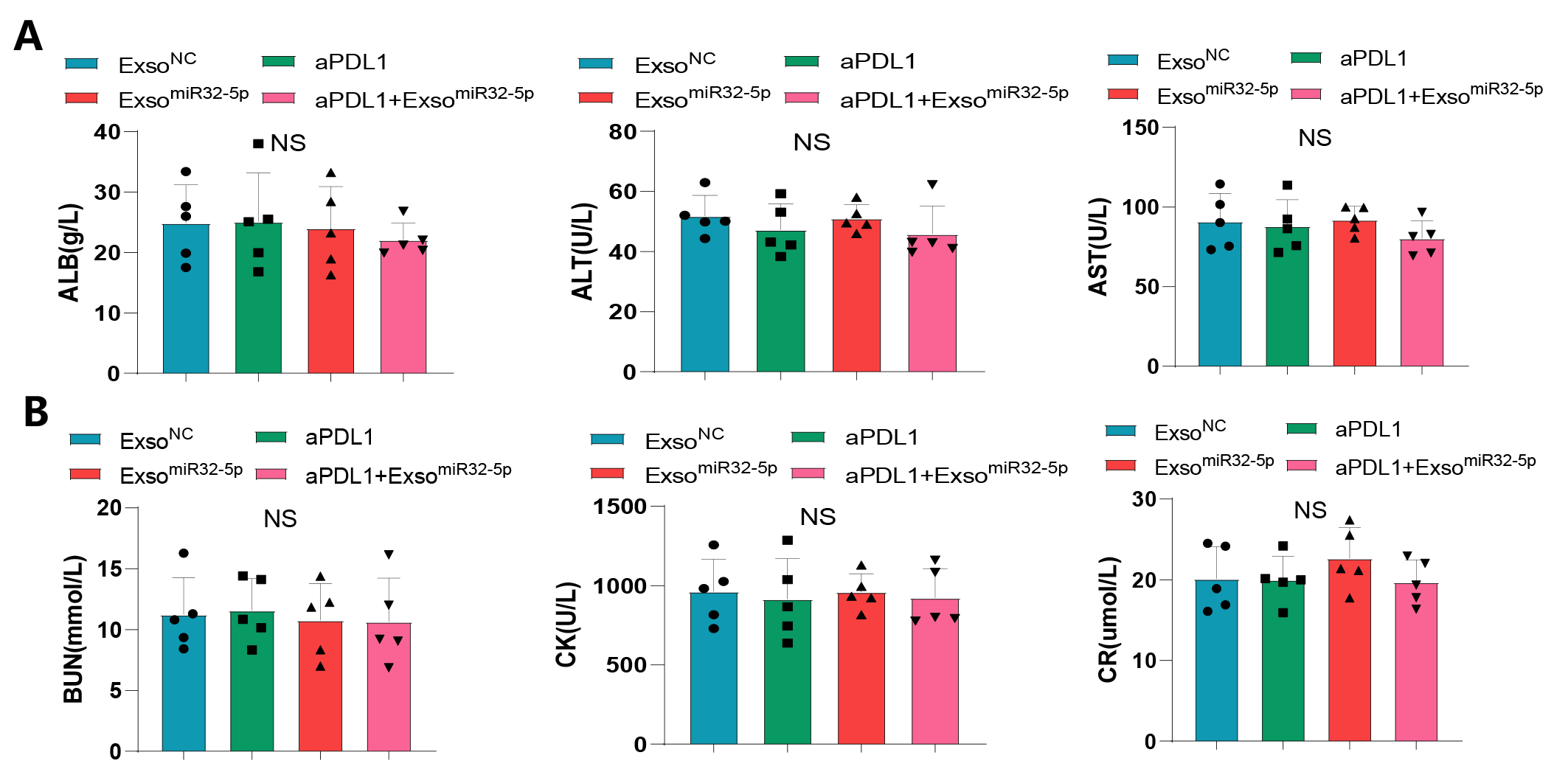

Supplement: Supplementary 1 — Figs. S1 to S5 [file research.1096.f1.zip › FigureS5.tif]
